# Factors Associated with Self-Reported Treatment Adherence During Pregnancy and Preliminary Psychometric Assessment of the PMAM in Primary Care

**DOI:** 10.3390/healthcare14193223

**Published:** 2026-09-30

**Authors:** Andrei Pănuș, Virginia Maria Rădulescu, Linda Nicoleta Bărbulescu, Marian Valentin Zorilă, Kamal Adina Maria, Gheorghe Gindrovel Dumitra, Denis Anamaria Mereț, Constantin Kamal Kamal

**Affiliations:** 1 Department of Urology, University of Medicine and Pharmacy of Craiova, 200349 Craiova, Romania; andrei.panus2@gmail.com; 2 Department of Medical Informatics, University of Medicine and Pharmacy of Craiova, 200349 Craiova, Romania; virginia.radulescu@umfcv.ro; 3 Department of Family Medicine, University of Medicine and Pharmacy of Craiova, 200349 Craiova, Romania; gindrovel.dumitra@umfcv.ro (G.G.D.); constantin.kamal@umfcv.ro (C.K.K.); 4 Department of Forensic Medicine, University of Medicine and Pharmacy of Craiova, 200349 Craiova, Romania; valentin.zorila@umfcv.ro; 5 Department of Internal Medicine, University of Medicine and Pharmacy of Craiova, 200349 Craiova, Romania; adina.kamal@umfcv.ro; 6 Faculty of Medicine, Transilvania University of Braşov, 500036 Braşov, Romania; denis.meret@unitbv.ro

**Keywords:** pregnancy, medication adherence, primary care, psychometrics, risk perception, clinician–patient communication, health literacy

## Abstract

**Background/Objectives:** Medication-taking behaviour during pregnancy may be associated with treatment-related beliefs, emotional concerns, information sources, and clinician–patient communication. We examined these cross-sectional associations and the preliminary measurement properties of the Pregnancy Medication Adherence Model (PMAM). **Methods:** This cross-sectional study included 355 pregnant women with prescribed treatment recruited from nine general practices in Dolj County, Romania. Q25 was analysed on its five-category scale and using an operational threshold of ≥4. Internal consistency, principal component analysis, an ordinal exploratory factor analysis based on polychoric correlations, regression modelling, and review-stage sensitivity analyses addressing criterion overlap were performed. **Results:** Overall, 255 participants (71.8%) scored ≥4 on Q25. The four multi-item domains had Cronbach’s α values of 0.897–0.935. Parallel analysis and principal-axis factoring with oblique rotation supported four factors; salient loadings were 0.797–0.925 and no secondary loading reached 0.30. In the original adjusted binary model, cognitive understanding, emotional reassurance, clinician–patient communication, and lower endorsement of information-seeking and social-influence items were associated with Q25 ≥ 4. After Q23 was removed from the communication score and Q19 and Q24 were omitted, these four associations persisted (adjusted ORs 1.90–2.55). **Conclusions:** The findings identify concurrent associations between medication-related perceptions and a single self-reported treatment-behaviour item. PMAM remains an exploratory framework; external psychometric evaluation and prospective assessment against defined medication-specific adherence and clinical outcomes are required before clinical use.

## 1. Introduction

Medication use during pregnancy is common and often necessary for the management of pre-existing conditions, acute illnesses, and pregnancy-related symptoms. Approximately half of pregnant women use at least one prescription medicine, and the proportion is higher when over-the-counter medicines, supplements, and herbal products are also considered [1,2].

Therapeutic decision-making during pregnancy requires careful consideration of maternal benefit, potential fetal risk, and the consequences of withholding necessary treatment. Pregnancy may, however, substantially alter medication-taking behaviour, even among women who previously followed their prescribed treatment. In a multinational study of pregnant women with chronic conditions, more than one-third of participants showed low adherence, with variation according to the underlying condition and individual beliefs about medicines [3].

Medication adherence in pregnancy is a multidimensional behaviour influenced not only by the treatment regimen itself, but also by beliefs about the necessity and safety of medicines. Some pregnant women prefer to avoid treatment altogether, even when it is medically indicated, which may result in failure to initiate therapy, dose reduction, or treatment discontinuation without consulting a clinician [3,4].

Fear of fetal harm is one of the main barriers to adherence. Pregnant women may overestimate the teratogenic risk of medicines while underestimating the risks associated with untreated maternal disease. Risk perception is influenced by educational attainment, previous experiences, the way information is communicated, and trust in the source of that information [4,5]. At the same time, limited health literacy may reduce a woman’s ability to understand the benefit-risk balance and may encourage misinterpretation of package leaflets or medical information [6].

The need for information about medication safety remains high throughout pregnancy. In addition to advice from healthcare professionals, pregnant women frequently consult the internet, social media, package leaflets, family members, and the experiences of other women [7]. Exposure to multiple, and sometimes conflicting, sources may heighten anxiety, reduce confidence in medical recommendations, and lead women to alter or discontinue treatment [8].

In this context, clinician–patient communication is essential. Adherence is more likely when a pregnant woman understands why a medicine has been prescribed, the expected benefits of treatment, the actual risks involved, and the consequences of non-treatment. Patients consistently seek information that is clear, individualised, and relevant to their own clinical circumstances, as well as opportunities to discuss concerns and information encountered online [9].

Primary care provides a particularly suitable setting for the early identification of cognitive, emotional, and social barriers because of continuity of care and repeated contact throughout pregnancy. However, evidence on interventions designed to improve medication adherence during pregnancy remains limited and heterogeneous, supporting the need for multidimensional assessment tools [10].

Previous studies have generally examined medication beliefs, risk perception, health literacy, information sources, and clinician communication as separate determinants of adherence. An integrated assessment may be particularly relevant in pregnancy, where these factors are likely to coexist and interact within the same treatment decision.

The primary objective was to examine cross-sectional associations between PMAM domain scores and self-reported treatment behaviour, assessed using Q25, after adjustment for demographic and obstetric characteristics. A secondary objective was to explore the internal consistency and dimensional structure of the questionnaire. The study was not designed to establish prospective prediction, causal pathways, or the clinical validity of a screening instrument.

## 2. Materials and Methods

### 2.1. Study Design and Participants

An observational, cross-sectional study was conducted to evaluate factors associated with medication adherence during pregnancy according to the conceptual framework of the Pregnancy Medication Adherence Model (PMAM). The study was carried out in nine general practice clinics in Dolj County, Romania, five located in urban areas and four in rural areas. Data were collected over a six-week period beginning in March 2026.

Pregnant women attending consultations during the study period were recruited consecutively. Eligibility required a confirmed pregnancy at any gestational stage, prescribed treatment during the current pregnancy, the ability to complete the questionnaire, and consent to participate. Of 482 women considered for inclusion, 127 had no prescribed treatment and were ineligible. All 355 eligible women agreed to participate and completed the questionnaire; no eligible participant declined and no questionnaire was excluded as incomplete or uninterpretable.

The analytical sample therefore comprised 355 pregnant women with prescribed treatment. Maternal age, area of residence, educational attainment, pregnancy trimester, and PMAM responses were recorded. The final dataset contained no missing values, and no imputation was performed. Participant-level practice identifiers and recruitment totals by practice were not retained in the analytical dataset.

### 2.2. PMAM Instrument and Data Collection

Data were collected using a structured, investigator-developed questionnaire comprising 24 scored items, Q5–Q28. The items represented cognitive, emotional, information-related, social, family, and clinician–patient aspects of medicine use during pregnancy. The present study provides a preliminary exploratory assessment of the questionnaire rather than validation of a clinical screening instrument. The available study documentation did not support reconstruction of a longer parent instrument or a formal short-form item-reduction process; the term ‘short form’ was therefore not retained. The questionnaire items were formulated by the investigator team with reference to previous research on medication perceptions and treatment adherence during pregnancy. The first 50 administrations were regarded by the investigators as a pilot phase and were retained in the analytical sample. The available documentation does not establish that a formal content-validity index or documented consensus procedure was undertaken. The exact four-domain composition was derived from the exploratory dimensional analyses rather than evaluated as a prespecified confirmatory structure.

Items Q5–Q24 assessed medication-related perceptions and potentially associated factors, whereas Q25–Q28 assessed treatment behaviours. Q25, ‘I followed the treatment as instructed’, was the primary outcome. Q26–Q28 addressed missed doses, discontinuation without medical approval, and avoidance of treatment initiation because of fear. Q29 was open-ended. The Romanian questionnaire is provided as Supplementary File S2 and its English translation as Supplementary File S3. No claim of formal cross-cultural validation is made for the translation.

Responses to Q5–Q24 were recorded from 1 (‘strongly disagree’) to 5 (‘strongly agree’); Q25–Q28 used anchors from 1 (‘never’) to 5 (‘always’). Questionnaires were completed during consultations without clinical staff influencing the responses; only administrative clarification was permitted. For Q25–Q28, participants were asked to consider any medication prescribed during the current pregnancy up to questionnaire completion. The reference period consequently varied with gestational age and was not medication-specific.

Participation was voluntary and anonymous. No directly identifying information was collected, and refusal to participate did not affect medical care. The study involved no clinical intervention, treatment modification, or collection of biological samples. The questionnaire included a statement explaining the voluntary nature of participation and indicating that completion of the questionnaire constituted agreement to participate.

### 2.3. Variables and Definition of Adherence

Maternal age was analysed as a continuous variable. Residence was coded as urban or rural; educational attainment was classified into five categories—lower secondary, upper secondary, post-secondary, university, and postgraduate—and gestational age was classified as first, second, or third trimester.

Q25 was analysed across its original five categories and as a binary outcome using Q25 ≥ 4. This threshold was an operational definition of a higher self-reported adherence response, not a clinically validated diagnostic cut-off. Scores below 4 indicate lower endorsement of Q25 and should not be equated with objectively verified non-adherence. The denominator for adherence analyses was the 355 participants with prescribed treatment.

### 2.4. PMAM Scores and Construct Structure

Item-level descriptive analyses used the raw responses. For composite-score calculation, negatively worded items—Q7, Q8, Q10–Q13, Q15–Q18, and Q24—were reverse-coded using the formula:

reverse-coded score = 6 − raw score

Q26–Q28 were reverse-coded when analysed as a secondary behavioural block. Composite scores were calculated as item means. For Q15–Q18, the reverse-coded score is described as lower endorsement of information-seeking and social-influence items. A higher score denotes less endorsement of the behaviours or influences described; it does not establish that these influences were inaccurate, harmful, or opposed to medical advice.

The final structure comprised seven constructs:

- Cognitive understanding and safety appraisal: Q5, Q6, Q7r, Q8r, and Q9.
- Emotional reassurance and lower fear: Q10r–Q13r and Q14.
- Lower endorsement of information-seeking and social-influence items: Q15r–Q18r.
- Family support: Q19.
- Trust and clinician–patient communication: Q20–Q23 in the original analysis; Q20–Q22 in the overlap sensitivity analysis.
- Lower unmet need for clarification: Q24r.
- Secondary behavioural non-adherence block: Q26r–Q28r.

Family support (Q19) and lower unmet need for clarification (Q24r) were single-item measures. Q26–Q28 were not entered into the original multivariable model because of their proximity to Q25. The six original explanatory scores were standardised as z-scores. Review-stage sensitivity analyses recalculated the communication score without Q23 and then omitted Q19 and Q24, because their wording also refers explicitly to following treatment.

### 2.5. Statistical Analysis

Data were entered and checked in Microsoft Excel 2021 (Microsoft Corporation, Redmond, WA, USA) and analysed initially using IBM SPSS Statistics, version 26.0 (IBM Corp., Armonk, NY, USA). Review-stage reproducibility and sensitivity analyses were independently conducted in Python 3.12 using pandas 2.2.3, NumPy 2.3.5, SciPy 1.17.0, and scikit-learn 1.8.0. Continuous and ordinal variables were summarised using means, standard deviations, medians, and interquartile ranges; categorical variables were reported as frequencies and percentages.

Distributional normality was assessed using the Shapiro–Wilk test and, where appropriate, the Kolmogorov–Smirnov test. Given the ordinal nature and non-normal distribution of the principal variables, inferential analyses relied predominantly on non-parametric methods.

Differences between urban and rural participants were assessed using the Mann–Whitney U test, whereas comparisons by educational attainment and pregnancy trimester were performed using the Kruskal–Wallis test. *p* Values from multiple item-level comparisons were adjusted using the Benjamini–Hochberg false-discovery-rate procedure. Effect sizes were expressed as rank-biserial correlation and epsilon-squared, as appropriate.

Internal consistency was evaluated using Cronbach’s alpha. The original dimensional reduction was reproduced as an unrotated principal component analysis of 18 explanatory items (Q5–Q18 and Q20–Q23) using Pearson correlations; Q19, Q24, and Q25–Q28 were excluded. KMO and Bartlett’s test assessed adequacy. In response to peer review, an ordinal exploratory factor analysis was added using a polychoric correlation matrix, principal-axis extraction, Promax oblique rotation, and parallel analysis with 50 independent permutations preserving item marginals. The number of factors was determined by parallel analysis and interpretability. Absolute loadings ≥ 0.40 were considered salient; secondary loadings ≥ 0.30 and a difference < 0.20 between the two largest loadings were flagged as potential cross-loadings. These review-stage criteria were not prespecified and the analysis remains exploratory.

The original binary logistic regression included six standardised PMAM scores, age, residence, and education and trimester entered as ordered covariates. To preserve transparency, this prespecified analysis was retained. Post-hoc review-stage models entered education and trimester categorically, recalculated clinician communication from Q20–Q22, and finally omitted Q19 and Q24. Linearity in the logit for age and continuous domain scores was assessed by jointly adding quadratic terms. Results are reported as adjusted odds ratios (aORs), 95% confidence intervals, and *p*-values. AUC in this cross-sectional study describes concurrent discrimination, not prospective prediction. A proportional-odds sensitivity model for the five Q25 categories was examined during revision, but numerical non-convergence and sparse outcome-by-education cells made the global assumption diagnostic unreliable; no ordinal regression coefficients are therefore interpreted. The lowest outcome category contained only 12 participants, including no Q25 = 1 observations in the postgraduate group. Supplementary Table S2 reports the relevant cell counts. More complex partial proportional-odds modelling was not pursued.

## 3. Results

### 3.1. Participant Characteristics and Self-Reported Adherence

Of 482 pregnant women considered, 127 were ineligible because they had no prescribed treatment. The final analysis included all 355 eligible women, each with complete data. Their mean age was 29.89 ± 4.91 years (median 30; range 18–42). Of these, 260 (73.2%) lived in urban areas and 95 (26.8%) in rural areas. Educational level and trimester distributions are shown in Table 1.

**Figure 1:**
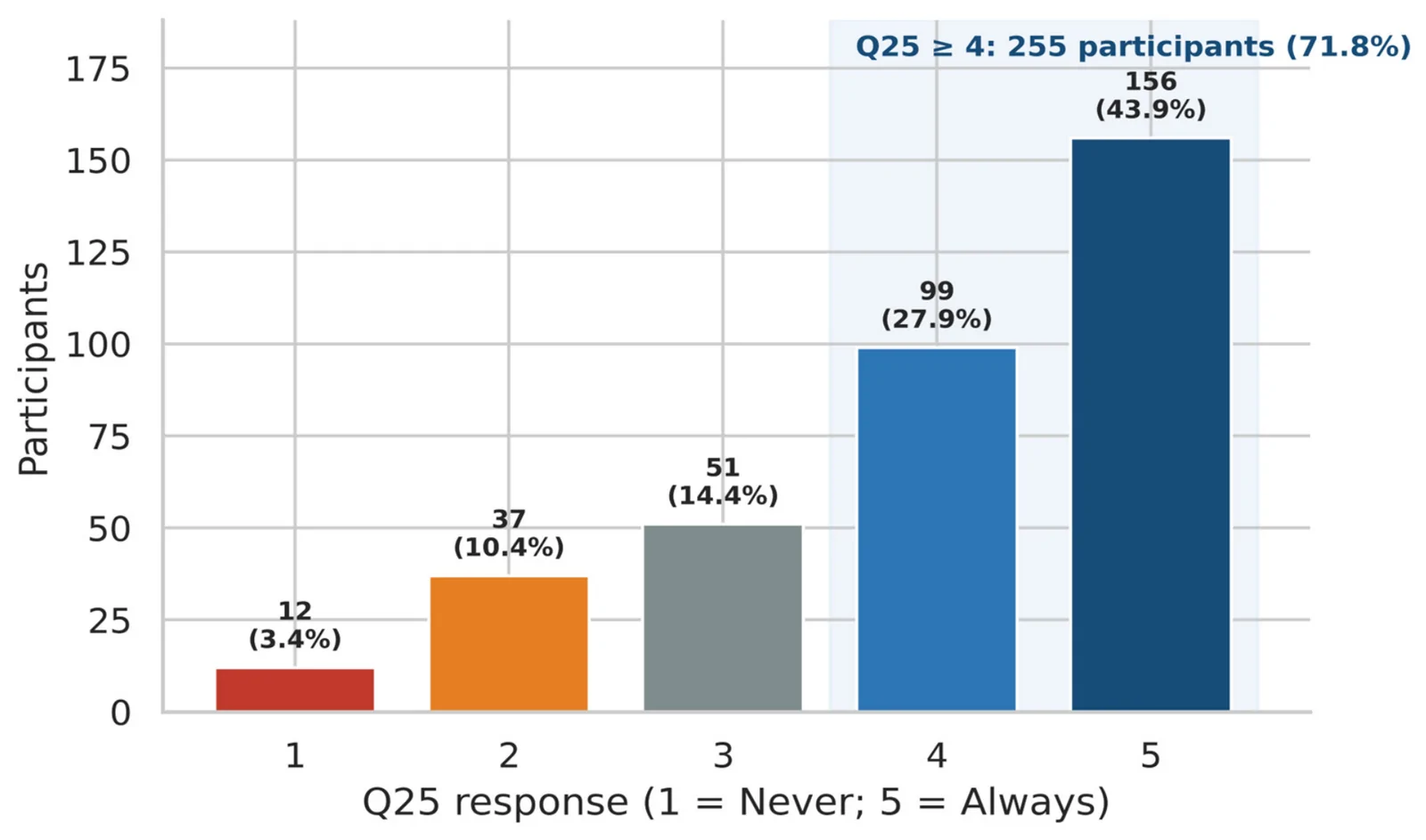
Distribution of Q25 responses. The operational threshold Q25 ≥ 4 included 255 participants (71.8%); it should not be interpreted as a clinically validated adherence cut-off. N = 355.

The mean Q25 score was 3.99 ± 1.14 and the median was 4 [3,4,5]. An operational Q25 score ≥ 4 was observed in 255 participants (71.8%); 100 participants (28.2%) scored below 4. Figure 1 shows all five response categories.

### 3.2. PMAM Item Responses and Psychometric Properties

At the item level, the most frequent barriers were fear that treatment might affect the baby, reported with scores of 4–5 by 201 participants (56.6%), and anxiety when taking medication, reported by 181 participants (51.0%). At the same time, 236 participants (66.5%) indicated a high level of trust in their physician, whereas 224 (63.1%) considered the treatment explanations to be clear. An additional need for clarification was expressed by 188 participants (53.0%). The full distribution of responses for Q5–Q28 is presented in Table S1.

**Figure 2:**
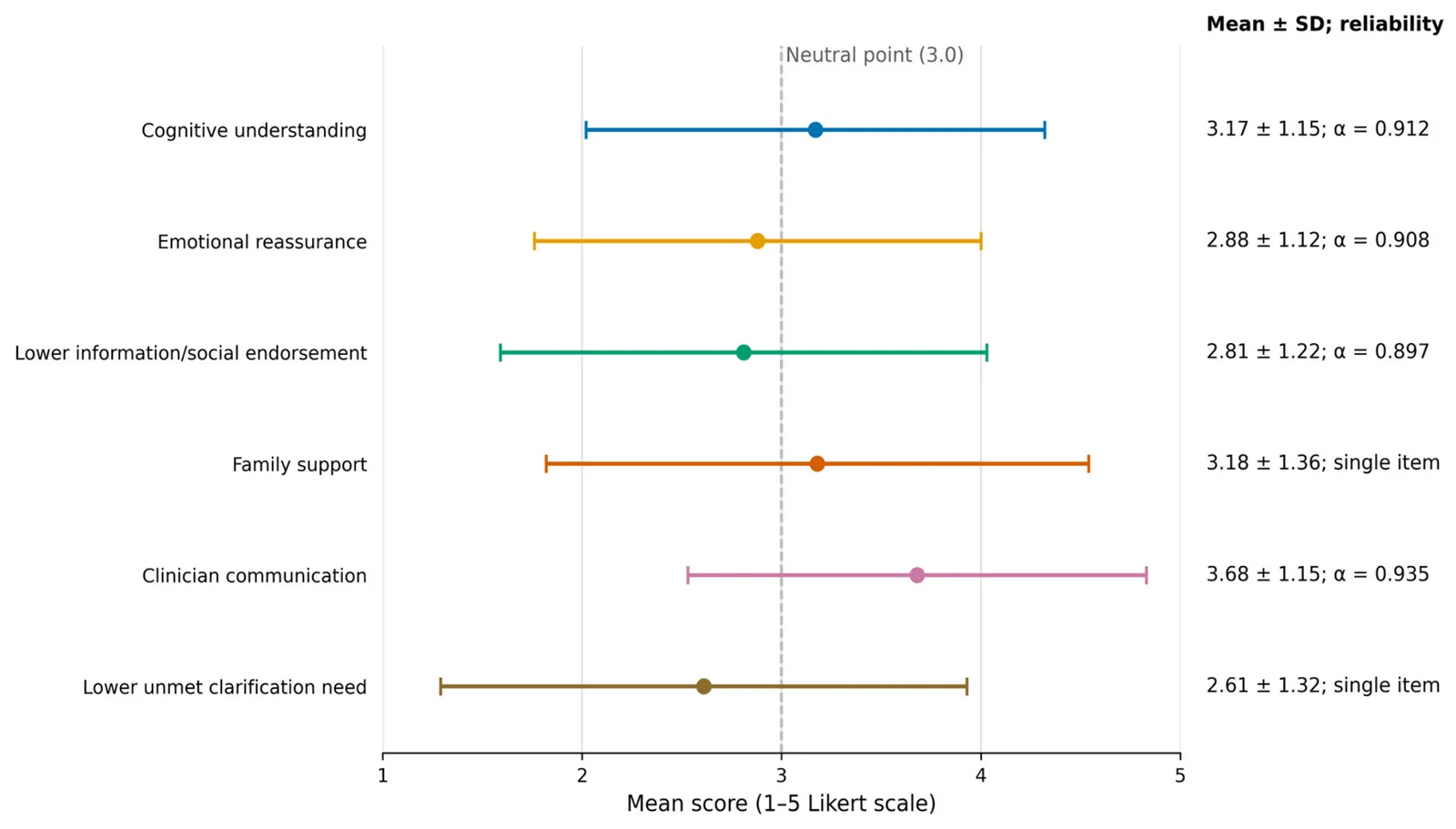
Mean PMAM scores with standard deviations. The Q15–Q18 score denotes lower endorsement of information-seeking and social-influence items; it is not interpreted as resilience or as an inherently favourable response. N = 355.

Among the PMAM dimensions, clinician–patient communication had the highest mean score (3.68 ± 1.15). Cognitive understanding and family support were slightly above the scale midpoint; emotional reassurance, lower endorsement of information-seeking and social-influence items, and lower unmet need for clarification were below 3. Cronbach’s alpha was 0.912 for the cognitive domain, 0.908 for the emotional domain, 0.897 for the information/social domain, and 0.935 for the original Q20–Q23 communication domain (Table 2 and Figure 2). After Q23 was removed, α remained 0.917 for Q20–Q22.

The reproduced unrotated PCA used 18 items, as indicated by Bartlett’s df = 153, and yielded KMO = 0.888, Bartlett’s χ^2^ = 4468.88, *p* < 0.001, and four components explaining 76.9% of variance. The ordinal exploratory factor analysis also retained four factors: the first four observed eigenvalues (6.563, 3.186, 2.628, and 2.249) exceeded the corresponding 95th-percentile values from parallel analysis (1.551, 1.434, 1.350, and 1.297), whereas the fifth did not (0.388 vs. 1.234). The four factors corresponded to emotional reassurance, clinician communication, lower information/social endorsement, and cognitive understanding. Salient pattern coefficients ranged from 0.797 to 0.925, communalities from 0.662 to 0.862, and no secondary loading reached 0.30 (Table 3). Factor correlations ranged from 0.170 to 0.420. These findings support an exploratory four-factor representation but do not establish content validity, confirmatory fit, or clinical validity.

### 3.3. Differences According to Sociodemographic and Obstetric Characteristics

After Benjamini–Hochberg adjustment, place of residence was associated with 23 of the 24 PMAM items, educational level with 16 items and pregnancy trimester with 8 items. The most consistent differences by residence involved cognitive, informational-social and clinician–patient relational items, whereas trimester-related differences were concentrated mainly in items pertaining to clinician–patient relationship and communication.

Self-reported Q25 responses were higher among urban than rural participants (means 4.23 ± 1.03 and 3.31 ± 1.18, respectively; U = 17,980, *p* < 0.001, r = −0.456). The operational Q25 ≥ 4 threshold was met by 209/260 urban participants (80.4%) and 46/95 rural participants (48.4%; χ^2^ = 35.13, *p* < 0.001). Educational level was also associated with Q25 (H = 20.15, df = 4, *p* < 0.001, ε^2^ = 0.057). Post-hoc differences involved postgraduate versus lower secondary, upper secondary versus postgraduate, and post-secondary versus postgraduate education.

**Figure 3:**
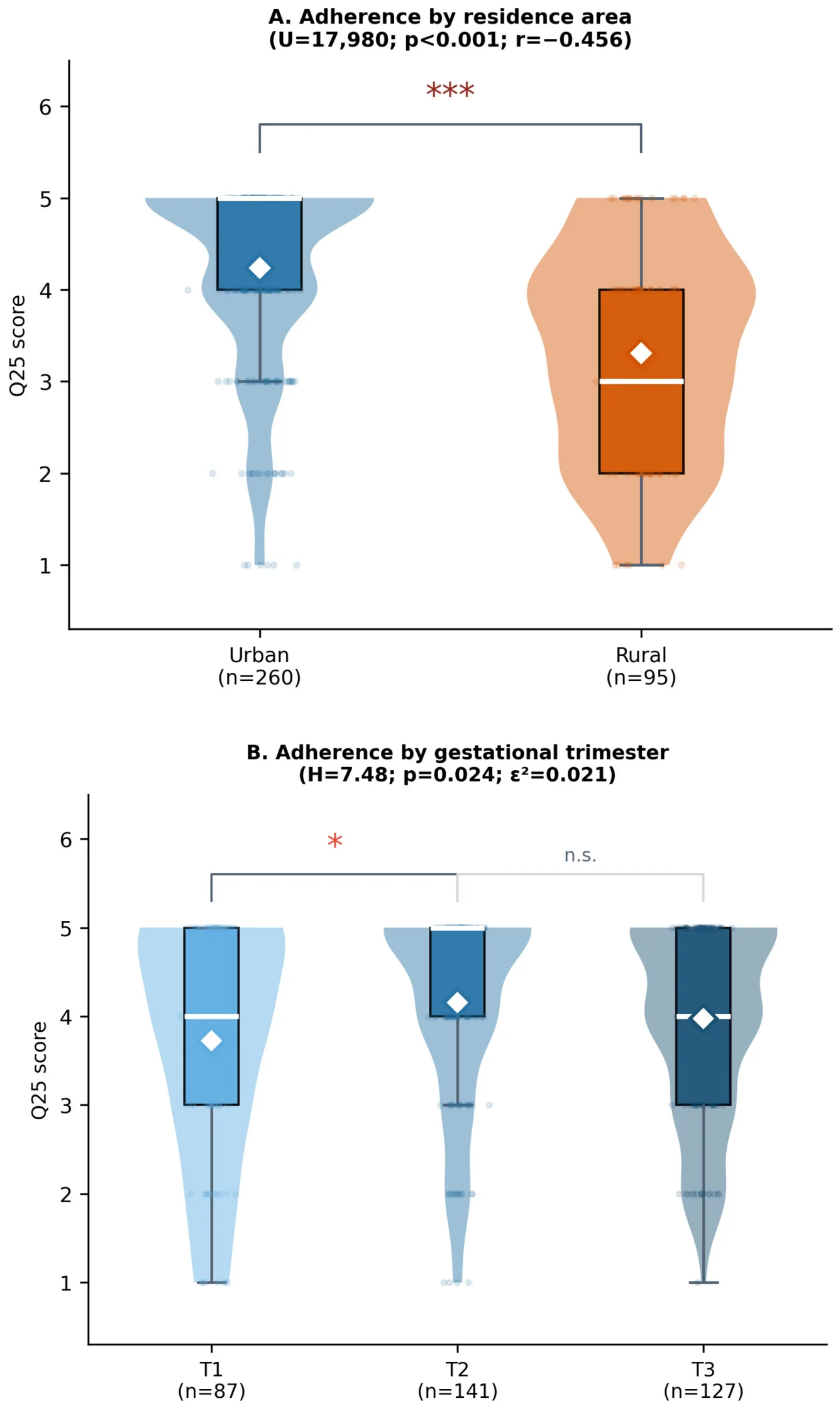
(**A**). Distribution of self-reported adherence Q25 according to place of residence. Diamonds indicate group means and horizontal lines indicate medians. N = 355. (**B**). Distribution of self-reported adherence Q25 according to pregnancy trimester. Diamonds indicate group means and horizontal lines indicate medians. N = 355. Significance annotations: * *p* < 0.05; *** *p* < 0.001; n.s. = not significant.

Differences across trimesters were observed for Q25 (H = 7.48, df = 2, *p* = 0.024, ε^2^ = 0.021), principally between the first and second trimesters (Bonferroni-adjusted *p* = 0.027). Q25 did not remain significant after false-discovery-rate correction within the item-level family (pFDR = 0.057). Maternal age correlated weakly with Q25 (ρ = 0.180, *p* < 0.001). These results are summarised in Table 4 and Figure 3A,B.

### 3.4. Bivariate Associations Between PMAM Dimensions and Adherence

All six original explanatory PMAM scores were positively correlated with Q25 (all *p* < 0.001). Emotional reassurance showed the largest correlation (ρ = 0.529), followed by clinician–patient communication (ρ = 0.489). Cognitive understanding, lower endorsement of information-seeking and social-influence items, family support, and lower unmet need for clarification showed weak-to-moderate correlations (ρ = 0.372–0.395; Table 5). Partial correlations are presented in Table S3 and Figure S1.

**Figure 4:**
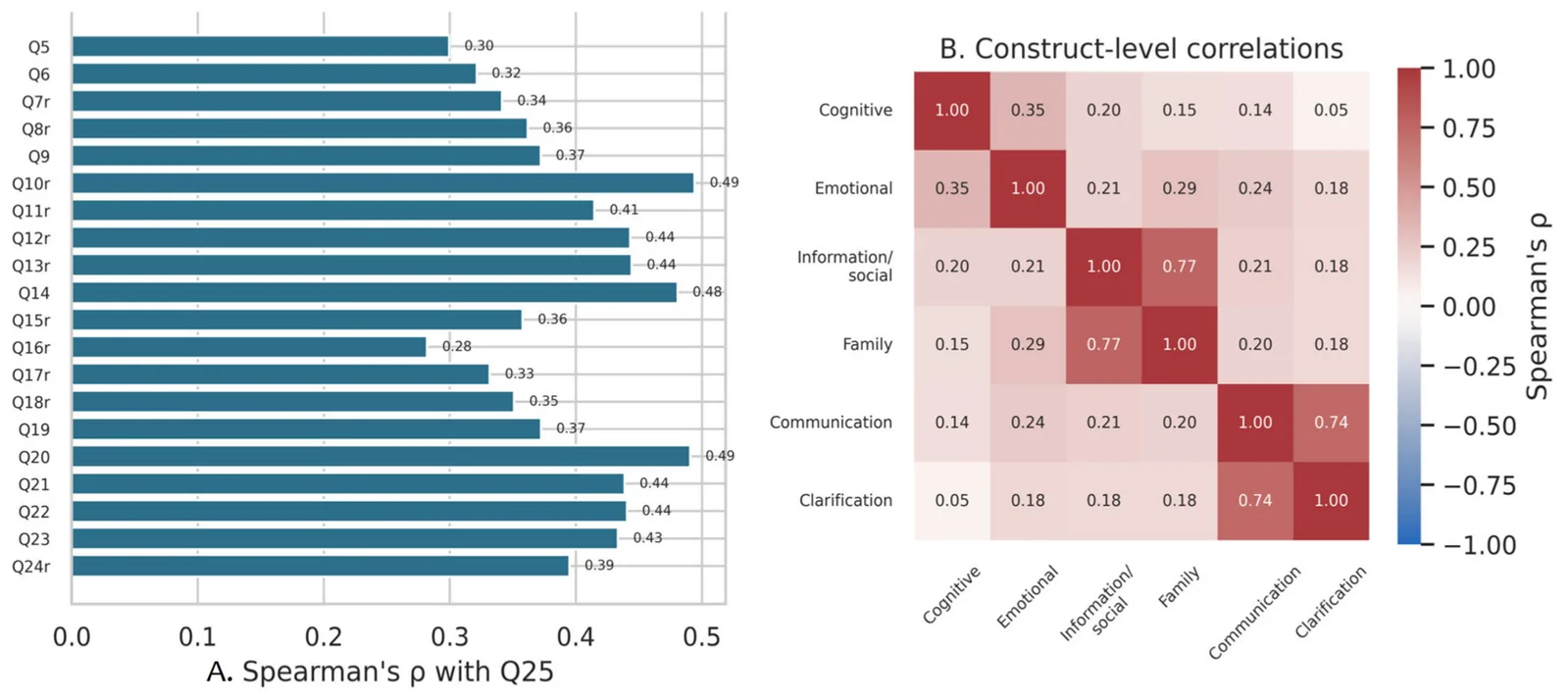
PMAM correlation structure: item-level associations with Q25 and correlations among the original explanatory scores. Reverse-coded items are marked ‘r’. N = 355. Panel A shows item-level associations with Q25; Panel B shows correlations among the original explanatory scores.

The behavioural block Q26–Q28 showed a strong correlation with Q25 (ρ = 0.776, *p* < 0.001). Given the conceptual proximity between these behaviours and the primary outcome, this block was not included in the multivariable model. At item level, the strongest associations with Q25 involved fear of fetal harm, trust in the physician, reassurance after the physician’s explanations, treatment avoidance, and the balance between fear and trust. The correlation structure at item and construct level is shown in Figure 4.

### 3.5. Multivariable Model and Discriminative Performance

The original binary logistic model was statistically significant (χ^2^(10) = 152.79, *p* < 0.001; Nagelkerke R^2^ = 0.503). The Hosmer–Lemeshow test showed no evidence of gross lack of fit (*p* = 0.688), and the maximum VIF was 2.54. Four domains were associated with the operational Q25 ≥ 4 outcome after mutual and demographic adjustment.

**Figure 5:**
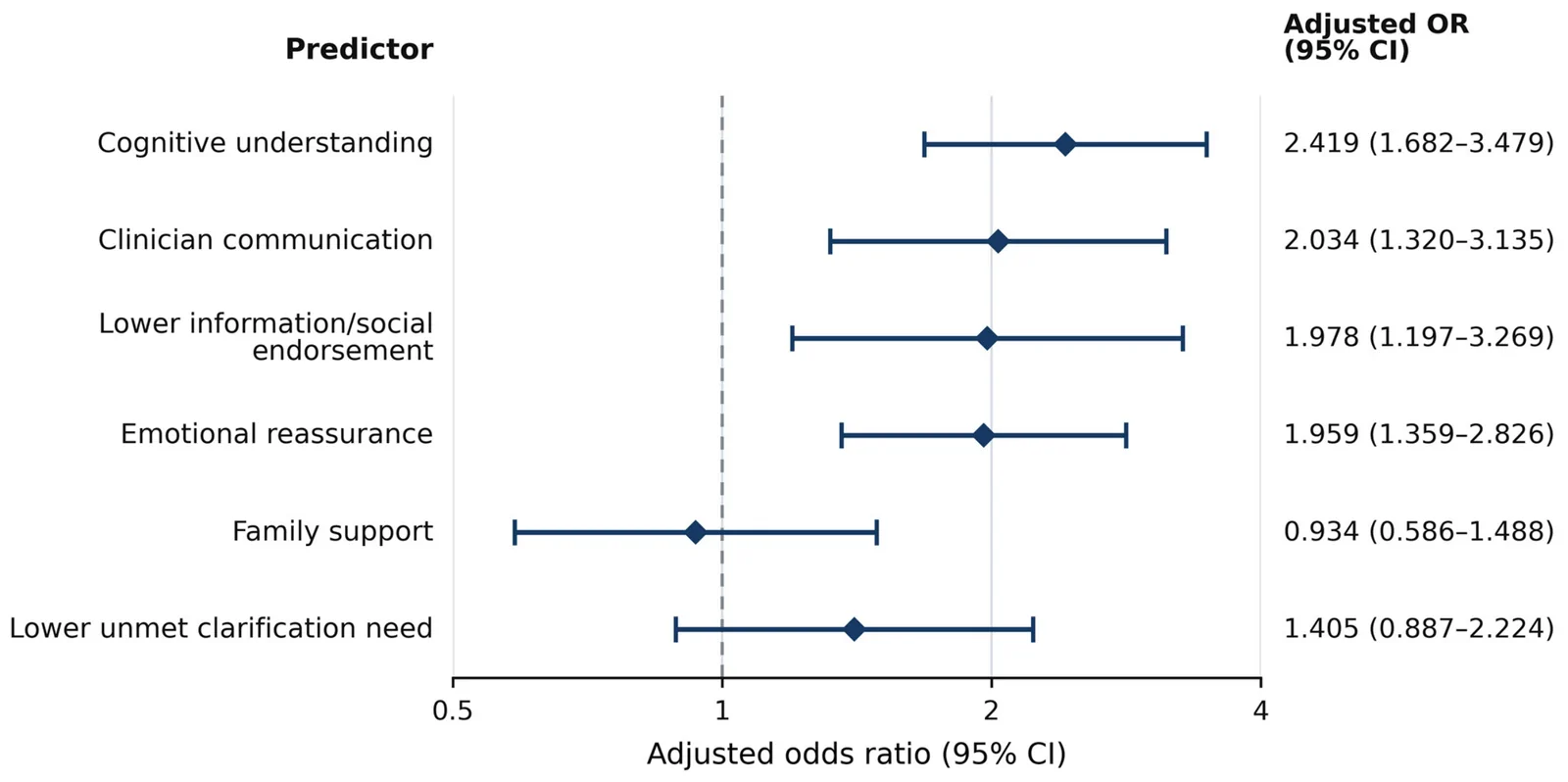
Adjusted associations of the original PMAM scores with Q25 ≥ 4. Odds ratios refer to a one-standard-deviation increase; horizontal bars show 95% confidence intervals. N = 355.

Per one-standard-deviation increase, cognitive understanding had the largest adjusted association (aOR = 2.419, 95% CI 1.682–3.479), followed by clinician communication (aOR = 2.034, 95% CI 1.320–3.135), lower endorsement of information-seeking and social-influence items (aOR = 1.978, 95% CI 1.197–3.269), and emotional reassurance (aOR = 1.959, 95% CI 1.359–2.826). Differences between point estimates do not establish a statistically tested hierarchy. Q19, Q24, and demographic covariates were not significant in this model (Table 6; Figure 5).

**Figure 6:**
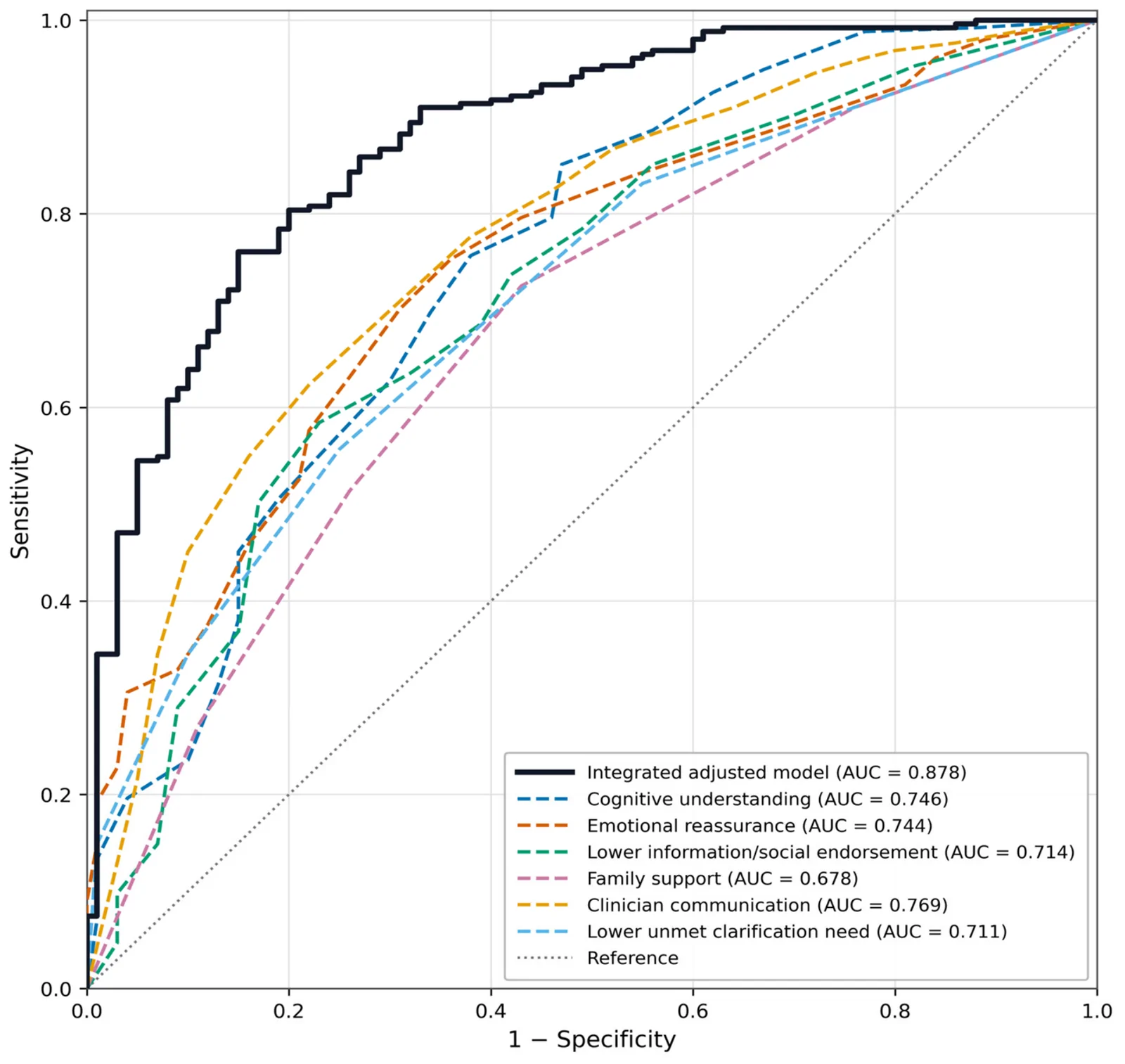
Concurrent ROC discrimination of Q25 ≥ 4 by the original integrated model and individual PMAM scores. AUC values do not represent prospective validation. N = 355.

The integrated model had an apparent AUC of 0.878. Bootstrap optimism correction yielded an AUC of 0.868 (95% CI 0.830–0.909), and 10-fold cross-validation yielded a mean AUC of 0.866 ± 0.064. These values quantify discrimination of concurrently measured Q25 responses within the same study context (Figure 6); they do not establish prospective predictive validity or transportability to other practices.

### 3.6. Review-Stage Sensitivity Analyses

Coding education and trimester categorically produced estimates similar to the original model. After Q23 was removed, the Q20–Q22 communication score retained high internal consistency (α = 0.917) and remained associated with Q25 ≥ 4 (aOR = 1.918, 95% CI 1.232–2.988, *p* = 0.004). In the more conservative model excluding Q19, Q23, and Q24, cognitive understanding (aOR = 2.293, 95% CI 1.585–3.318), emotional reassurance (aOR = 2.080, 95% CI 1.432–3.021), lower information/social endorsement (aOR = 1.903, 95% CI 1.320–2.742), and Q20–Q22 communication (aOR = 2.546, 95% CI 1.781–3.641) all remained associated with Q25 ≥ 4 (Table 7). Apparent AUC was 0.882 and Nagelkerke R^2^ was 0.514. Adding quadratic terms for the four scores and age did not improve fit jointly (χ^2^(5) = 8.58, *p* = 0.127). These post-hoc analyses reduce, but cannot eliminate, criterion overlap and common-method bias. The attempted proportional-odds sensitivity model was not retained because it showed numerical non-convergence and its global diagnostic was dominated by sparse outcome-by-education cells; the cell counts are shown in Supplementary Table S2.

### 3.7. Concerns Regarding Medication Use in Pregnancy

In the analysis of the open-ended Q29 responses, fear of fetal harm emerged as the dominant theme and was identified in 247 participants (69.6%). The influence of online information or other women’s experiences was reported by 59 participants (16.6%), insufficient communication or lack of information by 30 participants (8.5%), and general caution towards treatment by 19 participants (5.4%). Additional analyses are presented in Supplementary File S1: full item distributions in Table S1, the sparse-cell diagnostic for the attempted ordinal model in Table S2, partial correlations in Table S3 and Figure S1, threshold sensitivity in Table S4 and Figure S2, and internal resampling estimates for concurrent discrimination in Figure S3.

## 4. Discussion

### 4.1. Summary of the Principal Findings

Among 355 pregnant women with prescribed treatment, 255 (71.8%) scored ≥4 on a single self-reported treatment-behaviour item. This operational threshold is not an objectively verified adherence classification. The original and review-stage models consistently identified concurrent associations with cognitive understanding, emotional reassurance, clinician communication, and lower endorsement of information-seeking and social-influence items. Interpretation must nevertheless account for medication heterogeneity, variable recall periods, criterion overlap, common-method variance, and the unmodelled practice structure.

The study also shows that a generally favourable clinician relationship can coexist with persistent emotional concerns: 56.6% strongly endorsed fear that treatment could affect the fetus and 51.0% reported anxiety when taking medication. This pattern does not show that communication failed, nor that fear caused lower adherence. It indicates that understanding, reassurance, information exposure, and the therapeutic relationship were interrelated at the same assessment.

The review-stage models are particularly informative. Removing Q23 from the communication score and omitting Q19 and Q24 did not remove the four principal associations. This finding argues against complete explanation by direct wording overlap, although attenuation and shared self-report remain possible. Demographic attenuation after PMAM adjustment should not be interpreted as mediation; it may reflect shared variance, confounding, or model specification.

The apparent AUC of 0.878 and internally corrected estimates describe concurrent discrimination within this dataset. They do not establish prospective prediction, clinical risk stratification, or performance in other practices. PMAM should therefore be treated as an exploratory measurement framework, not a clinical screening instrument.

### 4.2. Adherence Levels in Relation to Previous Literature

The proportion scoring Q25 ≥ 4 (71.8%) lies within a plausible range reported in pregnancy pharmacotherapy studies, but direct comparison is limited by differences in conditions, medicines, exposure periods, and adherence measures. Here, Q25 was a single item referring to prescribed treatment during the pregnancy to date, and ≥4 was an operational threshold. Other studies have used multi-item scales, dispensing records, diaries, or biological indicators [11,12].

In a multinational study of pregnant women receiving treatment for chronic conditions, 36.2% showed low adherence, with marked variation by condition, from 17.1% in diabetes to 55.6% in rheumatic disease. Pregnancy-specific beliefs about medicines were key determinants of adherence [3]. Similarly, almost half of pregnant women taking psychotropic medication had low adherence, and higher perceived risk from antidepressants was associated with a greater likelihood of non-adherence [13]. These findings support the interpretation that the 28.2% identified in our study does not represent a marginal phenomenon, but rather reflects a recurring challenge across therapeutic contexts.

An important contribution of the present findings is that adherence was examined not merely as an endpoint, but through the dimensions that may support or impede it. Previous studies have shown that women who take medication for chronic disease tend to perceive its necessity and benefits more clearly and attribute less harmful potential to medicines than women who avoid treatment [14]. In a UK population, approximately three in ten women reported avoiding certain medicines during pregnancy, and stronger perceptions of risk were associated with leaving symptoms or conditions untreated [15]. In this context, PMAM adds an integrated perspective by examining cognition, emotion, social influence, and the therapeutic relationship simultaneously.

### 4.3. Fear of Fetal Harm and the Emotional Dimension

Fear of fetal harm was the most frequent barrier identified in both the quantitative and open-ended data. In Q29, 69.6% of participants directly described concerns about malformations, fetal toxicity, or unknown effects on the baby. The concordance between Q10 and the open-ended responses provides qualitative support for the clinical relevance of the emotional dimension and indicates that fear is a central element of therapeutic decision-making during pregnancy.

This finding is consistent with studies in which pregnant women expressed more restrictive attitudes towards medicines than towards other exposures and frequently overestimated teratogenic risk. In a study of 866 women in Norway, participants believed that medication use should be more restricted during pregnancy, with attitudes varying according to education and professional experience [4]. A study focusing on health literacy likewise showed that the ability to understand and use medical information was associated with perceptions of teratogenic risk and with health behaviours during pregnancy [6].

Emotional reassurance correlated with Q25 and remained associated across the original and review-stage binary models. This consistency does not establish that fear influences adherence causally. Reverse directionality is plausible, and longitudinal data are required to determine temporal order.

Qualitative evidence from other populations complements this interpretation. Interviews with pregnant women suggest that decisions about medicine use are often negotiated between concern for the baby, symptom severity, previous experiences, and trust in the source of advice [16]. In hypertensive disorders of pregnancy, women have described a tension between accepting the need for treatment and feeling vulnerable or lacking control over their own bodies [17]. An information-only intervention may therefore be insufficient when anxiety dominates decision-making. Counselling should acknowledge the concern, explicitly compare treatment risk with the risk of untreated disease, and check how the patient has understood the message.

### 4.4. Cognitive Understanding and the Necessity-Concerns Balance

Cognitive understanding and safety appraisal had the largest point estimate in the original binary model (aOR = 2.419 per standard deviation). This was not a formally tested ranking and should not be interpreted as a 2.4-fold increase in absolute probability. The result is consistent with an association between understanding treatment necessity and a higher Q25 response.

This finding is compatible with the Necessity-Concerns Framework, according to which adherence reflects an individual’s appraisal of treatment necessity relative to concerns about adverse effects. The meta-analysis by Horne and colleagues showed across a wide range of chronic conditions that stronger perceived necessity and fewer concerns are associated with better adherence [18]. During pregnancy, this balance becomes more complex because the woman evaluates potential consequences for both herself and the fetus. Even when the actual pharmacological risk is low, a risk perceived as uncontrollable or inadequately explained may outweigh the anticipated treatment benefit in the patient’s mind.

The PMAM findings suggest that counselling should include more than a statement that a medicine is permissible in pregnancy. It should also explain the therapeutic rationale, the potential severity of untreated disease, available alternatives, expected duration of treatment, and the degree of certainty in the evidence. Package leaflets may heighten fear through precautionary wording; 39.4% of participants reported that leaflet information made them consider the medicine dangerous. In addition, 41.1% reported difficulty distinguishing which medicines were safe. These findings support a teach-back approach, in which the clinician asks the patient to explain in her own words why she is taking the medicine and what risks have been discussed, so that misunderstandings can be identified before they lead to treatment interruption or avoidance.

Educational attainment was associated with Q25 in unadjusted analyses but not after multivariable adjustment. This attenuation does not demonstrate that education acts through cognitive or information-related competencies. Formal education should not be used as a proxy for health literacy; clinical assessment should focus directly on what the patient has understood.

### 4.5. The Clinician–Patient Relationship and the Role of Primary Care

The original Q20–Q23 clinician-communication score had the highest mean (3.68) and α = 0.935. Because Q23 explicitly refers to following treatment, it may share criterion content with Q25. When communication was recalculated from Q20–Q22, α remained 0.917, and the adjusted association persisted (aOR = 1.918, 95% CI 1.232–2.988). This supports robustness to the exclusion, but concurrent self-report and common-method variance remain; similar associations between clinician communication and adherence have been reported across other clinical contexts [19].

General practitioners are particularly well placed to integrate medical history, pregnancy progression, chronic disease, self-medication, and family context. Continuity of care makes it possible to revisit treatment decisions over repeated consultations, correct information obtained from unvalidated sources, and identify discontinuation at an early stage. The increase in relational scores from the first to the third trimester observed in our study may reflect progressive strengthening of the therapeutic alliance. However, because the study was cross-sectional, the same women were not followed over time, and trimester differences may also reflect differences between the groups assessed.

Interventions to improve medication adherence during pregnancy have been evaluated relatively infrequently and heterogeneously. A recent systematic review found that evidence for specific interventions remains limited, with substantial variation in populations, outcomes, and intervention strategies [10]. This gap supports the development of pragmatic primary-care interventions, such as a structured discussion of necessity and risk, a personalised written summary, verification of understanding, an agreed route for follow-up questions, and reassessment of adherence at subsequent visits. The PMAM domains may help generate hypotheses about potentially relevant counselling targets, but their usefulness for individualised intervention planning requires prospective evaluation.

### 4.6. Informational-Social Pressure, Rural Residence, and Education

The reverse-coded Q15–Q18 score was associated with Q25 in both the original and conservative models. Higher scores mean lower endorsement of online searching and social influences; they do not show that such information was inaccurate or that family and peer influence was harmful. These sources may be supportive, misleading, or mixed. The neutral label ‘lower endorsement of information-seeking and social-influence items’ therefore replaces ‘resilience’ throughout.

Online discussion can reveal concerns about medication safety, but it is not equivalent to pharmacological assessment [20]. Conflicting information can generate uncertainty and changes in treatment decisions [8], whilst family and peer sources may also provide useful support. The current questionnaire did not distinguish source quality, message direction, or whether advice conflicted with medical recommendations.

The corrected unadjusted contrast was 80.4% (209/260) in urban participants versus 48.4% (46/95) in rural participants. Residence was no longer associated after PMAM adjustment, but this attenuation is not evidence of mediation. Practice location and participant residence may be intertwined, and the absence of practice identifiers prevented assessment of clustering or practice-adjusted effects.

In practice, addressing misinformation should not be limited to warning patients that online sources may be unreliable. A more effective approach is to recommend verified resources proactively, explain how to assess the credibility of a website, and invite women to bring concerning online information into the consultation. In this way, the clinician does not compete with the digital environment solely through authority, but makes it part of the clinical dialogue.

### 4.7. Pregnancy Trimester, Age, and Demographic Factors

Adherence differed modestly across pregnancy trimesters, with the clearest contrast between the first and second trimesters; however, the effect was small and Q25 did not remain significant after FDR correction within the item-level family of tests. Because the comparison was cross-sectional, these differences should not be interpreted as within-person change over pregnancy and are better regarded as hypotheses for longitudinal assessment.

Maternal age and educational attainment showed bivariate associations with adherence but were not independently associated with the outcome in the fully adjusted model. Demographic characteristics may therefore identify population-level vulnerability but appear less informative for individual counselling than direct assessment of medication-related understanding, fear, and informational influences. Future longitudinal studies should examine how these PMAM dimensions evolve across pregnancy and how they relate to treatment changes, adverse effects, consultations, and information sources.

### 4.8. Contribution and Psychometric Status of the PMAM

The four multi-item scores had α values of 0.897–0.935. The reproduced PCA and the review-stage ordinal factor analysis both supported four dimensions. In the latter, all salient loadings were ≥0.797, no secondary loading reached 0.30, and parallel analysis supported four factors. This convergence is useful preliminary evidence for internal dimensionality, but it does not establish content validity, confirmatory fit, measurement invariance, stability over time, or criterion validity [21].

High internal consistency may reflect coherent domains, closely related wording, or redundancy. The questionnaire remains investigator-developed and exploratory; Q19 and Q24 are single items, and Q25 is not an external criterion. Independent confirmatory factor analysis, test–retest reliability, invariance testing, and comparison with established belief and adherence measures remain necessary.

The AUC estimates should be interpreted as concurrent discrimination of Q25 responses, not as validation of a prediction model. Resampling addresses internal optimism but cannot overcome criterion overlap, establish calibration in new populations, or demonstrate clinical utility.

### 4.9. Herbal Products and the Perception of “Natural” in Counselling During Pregnancy

The informational-social dimension of adherence should also encompass herbal and other non-prescription products. During pregnancy, the term “natural” may be intuitively associated with safety; in a multinational study of 9459 women, 28.9% reported using herbal products during pregnancy, most often through self-medication for pregnancy-related symptoms [22]. A subsequent analysis found that some products were contraindicated or required caution and highlighted limited human evidence on safety, dose, and interactions [23].

During antenatal consultations, medication histories should therefore explicitly include teas, extracts, supplements, and non-prescription products. Women should be encouraged to disclose their use without fear of disapproval, because plant origin does not remove the need to consider evidence, dose, purity, interactions, and pregnancy-specific safety. This approach may help prevent substitution of necessary treatment with insufficiently evaluated products.

### 4.10. Implications for Primary Care Practice

The findings may guide the content of future counselling research: clarifying why treatment is needed, eliciting the principal maternal or fetal concern, discussing information encountered outside the consultation, and checking understanding. They do not establish that PMAM-guided counselling improves adherence or health outcomes.

Clinically, adherence is beneficial only when the prescribed treatment is appropriate and when taking it as directed improves disease control or prevents complications. The size and nature of that benefit depend on the condition, medicine, dose, and untreated risk. This study did not measure maternal or fetal morbidity, hospitalisation, disease control, or adverse drug effects; it therefore cannot quantify a morbidity benefit from adherence. Those outcomes should be assessed prospectively alongside medication-specific adherence.

Because adherence may change with symptoms, adverse effects, pregnancy progression, online information, or family advice, medication-taking behaviour and concerns should be revisited at subsequent consultations in a neutral, non-judgemental manner.

### 4.11. Strengths and Limitations

Strengths include the sample size, consecutive recruitment across nine urban and rural practices, complete analytical data, multiple-comparison correction, effect-size reporting, and review-stage analyses addressing ordinal dimensionality and content overlap. The supplied Romanian questionnaire and its English translation also make the exact item wording and response anchors transparent. These features improve internal interpretability but do not remove limitations of concurrent self-report or unmodelled clustering.

The cross-sectional design does not establish temporal order or causality. All explanatory items and Q25 were measured concurrently. Q25 is a single self-reported item with an operational, non-validated threshold, and limitations of self-report should be considered [24,25]; recall and social-desirability bias are possible. The reference period covered the pregnancy to date and therefore varied by trimester. Responses could concern different medicines and durations. Q19, Q23, and Q24 contain wording close to adherence; sensitivity analyses reduce but cannot eliminate criterion overlap or common-method variance.

No objective adherence measure was collected. Medicine class, indication, disease severity, regimen complexity, duration, adverse effects, cost, and whether treatment was acute or chronic were not recorded. Having a prescription does not verify medicine use. The study also did not measure maternal or fetal morbidity, so clinical benefits of adherence cannot be estimated.

Participants were nested within nine practices, but practice identifiers and practice-specific recruitment totals were unavailable in the analytical dataset. Practice effects and within-practice dependence could therefore not be evaluated, which is particularly relevant to communication and urban–rural comparisons. Recruitment from one county, small outcome and education subgroups, and predominance of urban participants limit generalisability. Of 482 women considered, 127 lacked prescribed treatment and were ineligible; all 355 eligible women participated and completed the questionnaire. Complete participation among eligible women does not establish population representativeness. Q29 was coded post hoc by one evaluator.

### 4.12. Future Research

Future work should use a multicentre prospective design with repeated assessments and a clearly defined medication-specific reference period. PMAM should be administered before treatment initiation or change, and subsequent adherence should be measured using complementary self-report and objective indicators. Treatment class, indication, regimen complexity, disease control, maternal morbidity, hospitalisation, adverse effects, and fetal outcomes should be recorded so that predictive validity and clinical benefit can be evaluated directly.

One priority is to link self-reported adherence with pharmacy dispensing, pill counts, electronic monitoring, or other appropriate objective indicators and with prespecified maternal and fetal outcomes. Such data would distinguish concurrent questionnaire associations from prospective adherence and establish whether adherence to an indicated regimen translates into measurable clinical benefit.

Independent psychometric work should include confirmatory factor analysis, measurement invariance, test–retest reliability, convergent validity, and criterion validity against medication-use indicators. Any future prediction model should be evaluated in a separate dataset, with calibration and clinical utility assessed alongside discrimination.

A PMAM-guided intervention could then be tested against standard counselling. Outcomes should include medication-specific adherence, persistence, maternal disease control, anxiety, satisfaction, morbidity, and fetal safety. Until such evidence exists, the present domains are best regarded as hypotheses for counselling research rather than a clinical triage system.

## 5. Conclusions

Among pregnant women with prescribed treatment, cognitive understanding, emotional reassurance, clinician communication, and lower endorsement of information-seeking and social-influence items were concurrently associated with a higher Q25 response. These associations persisted after removing Q23 and omitting Q19 and Q24, but the cross-sectional design, single self-reported outcome, variable reference period, and absence of practice-level adjustment or objective adherence data constrain interpretation. The exploratory factor analysis supports four internally coherent domains; it does not establish a validated clinical instrument. An attempted proportional-odds analysis was not interpretable because of numerical and sparse-cell limitations. Prospective, medication-specific and externally validated research is required before PMAM can support screening, prediction, or claims of improved maternal or fetal outcomes.

## Figures and Tables

**Table 1:** Demographic and obstetric characteristics of the participants (N = 355).

Variable	Category/Statistic	*n* or Value	% or 95% CI
Age, years	Mean ± SD	29.89 ± 4.91	29.38–30.40
Age, years	Median [min–max]	30 [18–42]	—
Residence	Urban	260	73.2
Residence	Rural	95	26.8
Educational level	Lower secondary	20	5.6
Educational level	Upper secondary	119	33.5
Educational level	Post-secondary	54	15.2
Educational level	University	117	33.0
Educational level	Postgraduate	45	12.7
Pregnancy trimester	I	87	24.5
Pregnancy trimester	II	141	39.7
Pregnancy trimester	III	127	35.8

SD = standard deviation; CI = confidence interval; — = not applicable.

**Table 2:** PMAM scores and internal consistency of the constructs (N = 355).

Construct	Items	No. of Items	Mean ± SD	Median [IQR]	Cronbach’s α
Cognitive understanding and safety appraisal	Q5, Q6, Q7r, Q8r, Q9	5	3.17 ± 1.15	3.20 [2.20–4.20]	0.912
Emotional reassurance and lower fear	Q10r–Q13r, Q14	5	2.88 ± 1.12	2.80 [2.00–3.80]	0.908
Lower endorsement of information-seeking and social-influence items	Q15r–Q18r	4	2.81 ± 1.22	2.75 [1.75–3.75]	0.897
Family support	Q19	1	3.18 ± 1.36	3.00 [2.00–4.00]	—
Trust and clinician–patient communication	Q20–Q23	4	3.68 ± 1.15	4.00 [3.00–4.75]	0.935
Lower unmet need for clarification	Q24r	1	2.61 ± 1.32	2.00 [2.00–4.00]	—
Secondary behavioural block	Q26r–Q28r	3	3.05 ± 1.22	3.00 [2.00–4.00]	0.868

r = reverse-coded item; IQR = interquartile range; internal consistency cannot be estimated for single-item constructs.

**Table 3:** Pattern matrix from the ordinal exploratory factor analysis.

Item	Emotional	Communication	Information/Social	Cognitive	Communality
Q5	−0.016	−0.044	0.026	**0.855**	0.719
Q6	−0.042	0.067	−0.057	**0.912**	0.803
Q7r	0.039	0.040	−0.046	**0.842**	0.733
Q8r	−0.037	−0.012	0.114	**0.827**	0.711
Q9	0.076	−0.052	−0.019	**0.844**	0.751
Q10r	**0.876**	−0.016	0.007	−0.014	0.751
Q11r	**0.840**	−0.008	0.001	−0.000	0.702
Q12r	**0.863**	−0.081	−0.055	0.037	0.720
Q13r	**0.813**	0.037	0.062	−0.043	0.679
Q14	**0.849**	0.076	−0.004	0.025	0.781
Q15r	0.021	−0.003	**0.886**	0.048	0.817
Q16r	−0.075	−0.046	**0.878**	−0.015	0.723
Q17r	0.039	0.026	**0.798**	−0.009	0.662
Q18r	0.019	0.025	**0.884**	−0.037	0.788
Q20	0.003	**0.904**	0.008	0.006	0.825
Q21	0.028	**0.925**	−0.021	0.003	0.862
Q22	−0.025	**0.915**	0.004	0.013	0.831
Q23	−0.006	**0.909**	0.009	−0.008	0.826

Extraction: principal-axis factoring from polychoric correlations; rotation: Promax. Absolute loadings ≥ 0.40 are shown in bold. Factor signs are oriented for interpretability. No secondary loading reached 0.30.

**Table 4:** Associations of sociodemographic and obstetric characteristics with Q25.

Variable	Test	Descriptive Result	Statistic/Effect Size	*p*
Residence	Mann–Whitney U	Urban 4.23 ± 1.03; rural 3.31 ± 1.18	U = 17,980; r = −0.456	<0.001
High adherence by residence	χ^2^	Urban 80.4%; rural 48.4%	χ^2^ = 35.13	<0.001
Educational level	Kruskal–Wallis	Overall gradient across 5 levels	H = 20.15; ε^2^ = 0.057	<0.001
Pregnancy trimester	Kruskal–Wallis	T1 3.72; T2 4.16; T3 relatively stable	H = 7.48; ε^2^ = 0.021	0.024
Maternal age	Spearman	Weak positive association	ρ = 0.180	<0.001

**Table 5:** Spearman correlations between PMAM scores and Q25 (N = 355).

Dimension	Spearman’s ρ	*p*
Emotional reassurance and lower fear	0.529	<0.001
Trust and clinician–patient communication	0.489	<0.001
Lower unmet need for clarification	0.395	<0.001
Cognitive understanding and safety appraisal	0.387	<0.001
Lower endorsement of information-seeking and social-influence items	0.378	<0.001
Family support	0.372	<0.001
Secondary behavioural block Q26–Q28	0.776	<0.001

The Q26–Q28 block was excluded from the main logistic regression model because of conceptual overlap with Q25.

**Table 6:** Original binary logistic regression for the operational Q25 ≥ 4 outcome (N = 355).

Standardised Predictor	β	SE	OR	95% CI	*p*
Cognitive understanding and safety appraisal	0.883	0.185	2.419	1.682–3.479	<0.001
Trust and clinician–patient communication	0.710	0.219	2.034	1.320–3.135	0.001
Lower endorsement of information-seeking and social-influence items	0.682	0.256	1.978	1.197–3.269	0.008
Emotional reassurance and lower fear	0.673	0.183	1.959	1.359–2.826	<0.001
Family support	−0.068	0.238	0.934	0.586–1.488	0.775
Lower unmet need for clarification	0.340	0.234	1.405	0.887–2.224	0.147

Model adjusted for age, place of residence, educational level and pregnancy trimester. OR = odds ratio; CI = confidence interval. Overall model indicators: χ^2^(10) = 152.79, *p* < 0.001; Nagelkerke R^2^ = 0.503; AUC = 0.878; Hosmer–Lemeshow *p* = 0.688 (no evidence of gross lack of fit); maximum VIF = 2.54.

**Table 7:** Review-stage sensitivity analyses for Q25 ≥ 4.

Predictor	M1	M2	M3
Cognitive	2.444 (1.674–3.569); *p* < 0.001	2.409 (1.651–3.515); *p* < 0.001	2.293 (1.585–3.318); *p* < 0.001
Emotional	2.047 (1.405–2.982); *p* < 0.001	2.045 (1.404–2.980); *p* < 0.001	2.080 (1.432–3.021); *p* < 0.001
Lower information/social endorsement	1.957 (1.157–3.311); *p* = 0.012	1.969 (1.162–3.336); *p* = 0.012	1.903 (1.320–2.742); *p* < 0.001
Communication	1.952 (1.242–3.069); *p* = 0.004	1.918 (1.232–2.988); *p* = 0.004	2.546 (1.781–3.641); *p* < 0.001
Family support	0.968 (0.596–1.571); *p* = 0.895	0.961 (0.591–1.561); *p* = 0.871	Omitted
Lower unmet clarification need	1.620 (0.994–2.641); *p* = 0.053	1.650 (1.016–2.677); *p* = 0.043	Omitted
AUC	0.885	0.884	0.882
Nagelkerke R^2^	0.525	0.525	0.514

Values are adjusted odds ratios per standard deviation (95% CI). All models include age, residence, and education and trimester as categorical variables. M1 retains the original scores; M2 uses Q20–Q22 for communication; M3 additionally omits Q19 and Q24. AUC denotes concurrent discrimination.

## Data Availability

The individual-level data presented in this study are not publicly available because the participant information and consent process did not include permission for public deposition or unrestricted sharing of the data. Although direct identifiers were removed before analysis, the dataset contains combinations of sociodemographic and pregnancy-related variables that may entail a residual risk of participant re-identification. Access to a suitably de-identified dataset may be considered upon reasonable request to the corresponding author, subject to approval by the responsible institutional ethics committee and compliance with applicable data-protection requirements.

## Supplementary Materials

The following supporting information can be downloaded at: https://www.mdpi.com/article/10.3390/healthcare14193223/s1, Supplementary File S1, Statistical Supplementary Material, including Table S1: Descriptive statistics for raw PMAM responses; Table S2: Distribution of Q25 categories by educational attainment and sparse-cell diagnostic; Table S3: Bivariate and partial Spearman correlations with Q25; Table S4: Comparison of the primary and alternative Q25 thresholds; Figure S1: Bivariate and partial correlations; Figure S2: Threshold sensitivity; and Figure S3: Internal resampling estimates for concurrent discrimination. Supplementary File S2: Romanian-language PMAM questionnaire used in the study. Supplementary File S3: English translation of the Romanian-language questionnaire; this translation has not been separately validated.

## Abbreviations

The following abbreviations are used in this manuscript:

PMAM	Pregnancy Medication Adherence Model
AUC	Area under the receiver operating characteristic curve
CI	Confidence interval
FDR	False discovery rate
KMO	Kaiser-Meyer-Olkin
OR	Odds ratio
ROC	Receiver operating characteristic
VIF	Variance inflation factor
