# Supplementary material for: Factors Associated with Self-Reported Treatment Adherence During Pregnancy and Preliminary Psychometric Assessment of the PMAM in Primary Care
Source: Healthcare (Basel). 2026 Sep 30;14(19):3223. doi: 10.3390/healthcare14193223 (PMC13664985; doi:10.3390/healthcare14193223)
Supplement: Supplementary file 1 [file healthcare-14-03223-s001.zip › Supplementary File S1 - Statistical Material.pdf]

Statistical Supplementary Material

This file provides item-level distributions and secondary analyses that support the manuscript. Analyses are presented as descriptive or sensitivity analyses and do not convert the cross-sectional PMAM framework into a validated screening or prediction instrument.

S1. Additional statistical methods

Partial Spearman correlations were calculated after rank transformation and simultaneous adjustment for age, residence, educational attainment, and pregnancy trimester, with education and trimester entered as ordered covariates to reproduce the original analysis. Robustness to the operational binary threshold was examined by repeating the original logistic model with Q25 ≥ 3. Internal resampling comprised 500 bootstrap samples and 10-fold cross-validation; these estimates describe concurrent discrimination of Q25 within the study data, not prospective predictive validity.

A review-stage proportional-odds model was also attempted with Q25 retained on its five-category scale, education and trimester entered categorically, Q20–Q22 used for communication, and Q19, Q23, and Q24 excluded. The model showed numerical non-convergence. The global parallel-lines diagnostic was dominated by sparse outcome-by-education cells, particularly the absence of Q25 = 1 observations in the postgraduate group. Because the lowest Q25 category contained only 12 observations, ordinal coefficients and a partial proportional-odds extension were not retained. Table S2 documents the relevant cell structure.

S2. Full distribution of PMAM responses

Table S1. Descriptive statistics for raw responses to PMAM items Q5–Q28 (N = 355)

Item	Domain	Abbreviated wording	Dir.	Mean ± SD	Median [IQR]	1–2, n (%)	3, n (%)	4–5, n (%)
Q5	Cognitive	I understand why the treatment was prescribed	+	3.44 ± 1.27	4 [2–4]	91 (25.6)	69 (19.4)	195 (54.9)
Q6	Cognitive	I understand the benefits of treatment	+	3.26 ± 1.37	4 [2–4]	118 (33.2)	59 (16.6)	178 (50.1)
Q7	Cognitive	Patient leaflets make me think the treatment is dangerous	−	3.10 ± 1.31	3 [2–4]	132 (37.2)	83 (23.4)	140 (39.4)
Q8	Cognitive	I find it difficult to distinguish safe medicines	−	2.99 ± 1.40	3 [2–4]	147 (41.4)	62 (17.5)	146 (41.1)
Q9	Cognitive	I understand the risks of not taking treatment	+	3.25 ± 1.33	3 [2–4]	111 (31.3)	67 (18.9)	177 (49.9)
Q10	Emotional	I am afraid the treatment may affect the baby	−	3.48 ± 1.31	4 [2–5]	96 (27.0)	58 (16.3)	201 (56.6)
Q11	Emotional	I feel anxious when taking medicines	−	3.38 ± 1.34	4 [2–5]	107 (30.1)	67 (18.9)	181 (51.0)
Q12	Emotional	I prefer to avoid treatments	−	2.93 ± 1.30	3 [2–4]	142 (40.0)	85 (23.9)	128 (36.1)
Q13	Emotional	Fear is stronger than trust	−	3.08 ± 1.32	3 [2–4]	135 (38.0)	60 (16.9)	160 (45.1)
Q14	Emotional	I feel calmer after the doctor's explanations	+	3.30 ± 1.29	3 [2–4]	107 (30.1)	78 (22.0)	170 (47.9)
Q15	Information/social	I look up information about medicines on the internet	−	3.25 ± 1.36	3 [2–4]	119 (33.5)	73 (20.6)	163 (45.9)
Q16	Information/social	Social media influence my decisions	−	3.08 ± 1.41	3 [2–4]	138 (38.9)	63 (17.7)	154 (43.4)
Q17	Information/social	My family influences my decisions	−	3.24 ± 1.43	3 [2–5]	124 (34.9)	55 (15.5)	176 (49.6)
Q18	Information/social	The experiences of other pregnant women matter to me	−	3.18 ± 1.37	3 [2–4]	127 (35.8)	70 (19.7)	158 (44.5)
Q19	Family support	My family's support helps me follow treatment	+	3.18 ± 1.36	3 [2–4]	127 (35.8)	71 (20.0)	157 (44.2)
Q20	Communication	I trust my doctor	+	3.76 ± 1.24	4 [3–5]	66 (18.6)	53 (14.9)	236 (66.5)
Q21	Communication	My doctor explained the treatment clearly	+	3.68 ± 1.25	4 [3–5]	74 (20.8)	57 (16.1)	224 (63.1)
Q22	Communication	I felt listened to	+	3.63 ± 1.32	4 [3–5]	77 (21.7)	63 (17.7)	215 (60.6)
Q23	Communication	My relationship with the doctor helps me follow treatment	+	3.66 ± 1.22	4 [3–5]	67 (18.9)	73 (20.6)	215 (60.6)
Q24	Clarification	Clearer explanations would help me follow treatment	−	3.39 ± 1.32	4 [2–4]	98 (27.6)	69 (19.4)	188 (53.0)

Item	Domain	Abbreviated wording	Dir.	Mean ± SD	Median [IQR]	1–2, n (%)	3, n (%)	4–5, n (%)
Q25	Treatment behaviour	I followed treatment as instructed	+	3.99 ± 1.14	4 [3–5]	49 (13.8)	51 (14.4)	255 (71.8)
Q26	Treatment behaviour	I forgot to take my medicines	−	3.06 ± 1.34	3 [2–4]	140 (39.4)	68 (19.2)	147 (41.4)
Q27	Treatment behaviour	I stopped treatment without medical agreement	−	3.00 ± 1.35	3 [2–4]	142 (40.0)	63 (17.7)	150 (42.3)
Q28	Treatment behaviour	I avoided starting treatment because of fear	−	3.08 ± 1.42	3 [2–4]	135 (38.0)	63 (17.7)	157 (44.2)

Dir. = item direction. For negatively worded items, raw scores of 4–5 indicate stronger endorsement of the barrier or non-adherence behaviour; reverse coding was used only for composite scores. IQR = interquartile range.

S3. Ordinal outcome diagnostic

The five-category outcome was markedly sparse in its lowest category. The attempted proportional-odds model did not provide sufficiently stable diagnostics for inferential reporting. The table below is included to make that limitation auditable rather than to imply that the assumption was satisfied.

Table S2. Distribution of Q25 response categories by educational attainment (N = 355)

Educational attainment	Q25 = 1	Q25 = 2	Q25 = 3	Q25 = 4	Q25 = 5	Total
Lower secondary	2	6	3	3	6	20
Upper secondary	5	14	17	40	43	119
Post-secondary	1	6	14	15	18	54
University	4	8	11	34	60	117
Postgraduate	0	3	6	7	29	45
Total	12	37	51	99	156	355

No participant with postgraduate education selected Q25 = 1. Only 12 participants selected Q25 = 1 overall. The attempted model also showed numerical non-convergence; ordinal odds ratios are therefore not reported.

S4. Partial correlations

After adjustment for age, residence, educational attainment, and trimester, all six original PMAM scores remained associated with Q25 (all $p < 0.001$). These are concurrent rank associations and should not be interpreted as causal pathways.

Table S3. Bivariate and partial Spearman correlations with Q25 (N = 355)

Construct	Bivariate ρ	Partial ρ	Δ	95% CI for partial ρ	p
Cognitive understanding	0.387	0.289	−0.098	0.190–0.380	<0.001
Emotional reassurance	0.529	0.501	−0.028	0.420–0.580	<0.001
Lower endorsement of information-seeking and social-influence items	0.378	0.249	−0.129	0.150–0.350	<0.001
Family support	0.372	0.278	−0.094	0.180–0.370	<0.001
Clinician–patient communication	0.489	0.412	−0.077	0.320–0.490	<0.001
Lower unmet need for clarification	0.395	0.324	−0.071	0.230–0.410	<0.001

Partial correlations were adjusted simultaneously for age, residence, education, and trimester using rank-transformed variables. Confidence intervals are the intervals reported in the original supplementary analysis.

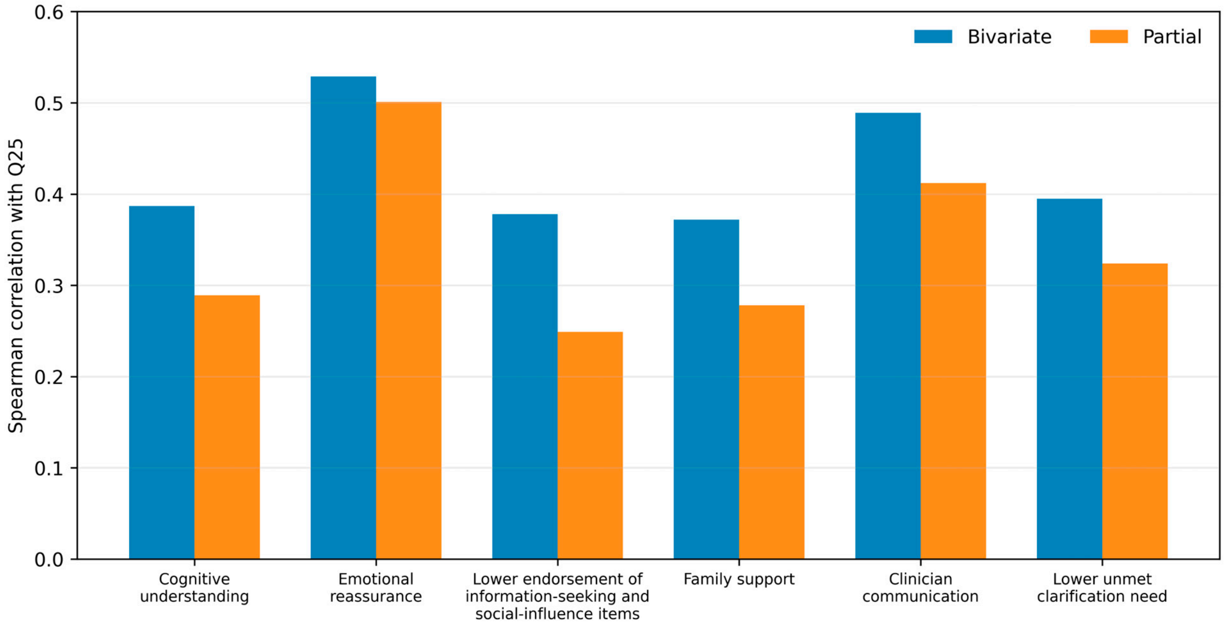

Figure S1. Comparison of bivariate and partial correlations between the PMAM scores and Q25. Partial correlations were adjusted for age, residence, education, and trimester.

S5. Sensitivity to the operational Q25 threshold

Using the more permissive threshold Q25 ≥ 3 yielded Nagelkerke R^2 = 0.503, AUC = 0.906, and Hosmer–Lemeshow p = 0.879. The same four multi-item scores remained associated in the same direction. For the score representing lower endorsement of information-seeking and social-influence items, the lower confidence limit was 1.001 and p = 0.050, indicating borderline precision rather than stronger evidence.

Table S4. Comparison of original-model results using Q25 ≥ 4 and Q25 ≥ 3 (N = 355)

Predictor	OR ≥ 4	95% CI ≥ 4	p ≥ 4	OR ≥ 3	95% CI ≥ 3	p ≥ 3
Cognitive understanding	2.419	1.682–3.479	<0.001	2.005	1.284–3.129	0.002
Emotional reassurance	1.959	1.359–2.826	<0.001	2.195	1.322–3.645	0.002
Lower endorsement of information-seeking and social-influence items	1.978	1.197–3.269	0.008	2.001	1.001–3.999	0.050
Family support	0.934	0.586–1.488	0.775	1.097	0.596–2.019	0.767
Clinician–patient communication	2.034	1.320–3.135	0.001	2.493	1.381–4.500	0.002
Lower unmet need for clarification	1.405	0.887–2.224	0.147	1.507	0.742–3.059	0.256

Odds ratios are per one-standard-deviation increase and come from the original adjusted specification. Thresholds are operational, not clinically validated cut-offs.

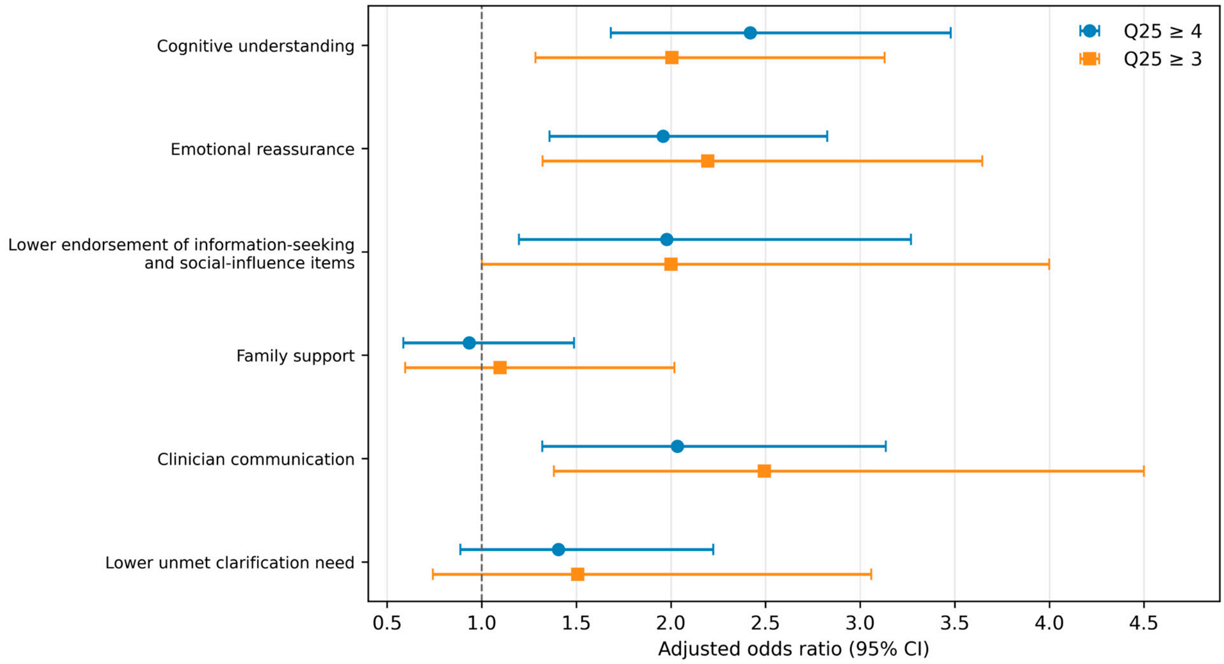

Figure S2. Sensitivity of adjusted associations to the operational threshold used for Q25. Symbols show odds ratios and horizontal bars show 95% confidence intervals.

S6. Internal resampling of concurrent discrimination

The apparent AUC was 0.878. Bootstrap-estimated optimism was 0.0097, giving an optimism-corrected AUC of 0.868 (95% CI 0.830–0.909). Ten-fold cross-validation yielded a mean AUC of 0.866 ± 0.064. These estimates quantify internal stability for discrimination of a concurrently measured Q25 response. They do not establish prospective prediction, external validity, calibration in another population, or clinical utility.

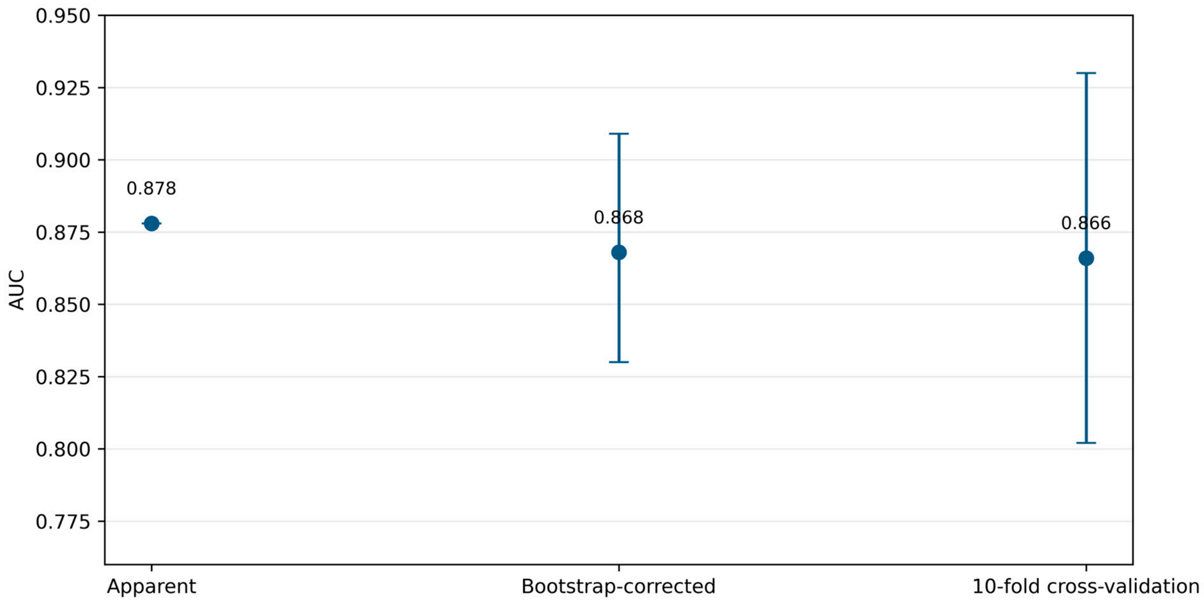

Figure S3. Apparent AUC and internal resampling estimates. The bootstrap bar represents the reported 95% confidence interval; the cross-validation bar represents ±1 SD across folds.
