# Supplementary material for: Factors Associated with Self-Reported Treatment Adherence During Pregnancy and Preliminary Psychometric Assessment of the PMAM in Primary Care
Source: Healthcare (Basel). 2026 Sep 30;14(19):3223. doi: 10.3390/healthcare14193223 (PMC13664985; doi:10.3390/healthcare14193223)
Supplement: Supplementary file 1 [file healthcare-14-03223-s001.zip › Supplementary File S2 Romanian questionnaire.pdf]

Pregnancy Medication Adherence Model (PMAM)
Chestionar în limba română

Formular pentru material suplimentar

Acest chestionar evaluează percepțiile, preocupările, influențele informaționale și sociale, relația cu medicul și comportamentele legate de administrarea medicamentelor în timpul sarcinii. Răspunsurile sunt confidențiale și sunt utilizate în scop de cercetare.

Participarea la acest studiu este voluntară. Prin completarea chestionarului, confirmați că ați fost informat(ă) cu privire la scopul studiului și că sunteți de acord să participați.

Secțiunea A. Date sociodemografice și obstetricale

Vârsta: ________ ani

Mediul de proveniență: ☐ Urban ☐ Rural

Nivelul de educație: ☐ Gimnazial ☐ Liceal ☐ Postliceal ☐ Universitar ☐ Postuniversitar

Trimestrul sarcinii: ☐ I ☐ II ☐ III

Secțiunea B. Percepții și factori asociați utilizării medicamentelor în sarcină – Q5–Q24

Scala de răspuns: 1 = Total dezacord … 5 = Total acord

Item	1	2	3	4	5
Q5. Înțeleg de ce mi-a fost prescris tratamentul.	☐	☐	☐	☐	☐
Q6. Înțeleg beneficiile tratamentului.	☐	☐	☐	☐	☐
Q7. Prospectele mă fac să cred că tratamentul este periculos.	☐	☐	☐	☐	☐
Q8. Îmi este greu să diferențiez medicamentele sigure.	☐	☐	☐	☐	☐
Q9. Înțeleg riscurile neadministrării tratamentului.	☐	☐	☐	☐	☐
Q10. Mi-e teamă că tratamentul afectează copilul.	☐	☐	☐	☐	☐
Q11. Mă simt anxioasă când iau medicamente.	☐	☐	☐	☐	☐
Q12. Prefer să evit tratamentele.	☐	☐	☐	☐	☐
Q13. Frica este mai puternică decât încrederea.	☐	☐	☐	☐	☐
Q14. Mă simt mai liniștită după explicațiile medicului.	☐	☐	☐	☐	☐
Q15. Caut informații despre medicamente pe internet.	☐	☐	☐	☐	☐
Q16. Rețelele sociale îmi influențează deciziile.	☐	☐	☐	☐	☐
Q17. Familia îmi influențează deciziile.	☐	☐	☐	☐	☐
Q18. Experiențele altor gravide contează pentru mine.	☐	☐	☐	☐	☐
Q19. Sprijinul familiei mă ajută să respect tratamentul.	☐	☐	☐	☐	☐
Q20. Am încredere în medic.	☐	☐	☐	☐	☐
Q21. Medicul mi-a explicat clar tratamentul.	☐	☐	☐	☐	☐
Q22. M-am simțit ascultată.	☐	☐	☐	☐	☐

Item	1	2	3	4	5
Q23. Relația cu medicul mă ajută să respect tratamentul.	☐	☐	☐	☐	☐
Q24. Explicații mai clare m-ar ajuta să urmez tratamentul.	☐	☐	☐	☐	☐

Secțiunea C. Aderență și comportamente de nonaderență – Q25–Q28

Scala de răspuns: 1 = Niciodată … 5 = Întotdeauna

Perioada de referință pentru Q25–Q28: luați în considerare orice medicament prescris în cursul sarcinii actuale, până la momentul completării chestionarului.

Item	1	2	3	4	5
Q25. Am urmat tratamentul conform indicațiilor.	☐	☐	☐	☐	☐
Q26. Am uitat să iau medicamentele.	☐	☐	☐	☐	☐
Q27. Am întrerupt tratamentul fără acord medical.	☐	☐	☐	☐	☐
Q28. Am evitat să încep tratamentul din teamă.	☐	☐	☐	☐	☐

Secțiunea D. Întrebare deschisă

Q29. Care este principala dumneavoastră teamă legată de administrarea medicamentelor în sarcină?

____

____

____

____

____

Notă metodologică

Notă: Q5–Q24 evaluează percepții și factori potențial asociați aderenței. Q25 reprezintă itemul principal de aderență auto-raportată, iar Q26–Q28 evaluează comportamente asociate nonaderenței. Q29 este o întrebare deschisă. Itemii formulați negativ sunt recodați invers numai pentru calcularea scorurilor compozite; răspunsurile brute se păstrează în formular. Acest document reproduce chestionarul utilizat în studiu; includerea sa ca material suplimentar nu reprezintă, prin ea însăși, validarea unui instrument clinic.
