# Supplementary material for: Factors Associated with Self-Reported Treatment Adherence During Pregnancy and Preliminary Psychometric Assessment of the PMAM in Primary Care
Source: Healthcare (Basel). 2026 Sep 30;14(19):3223. doi: 10.3390/healthcare14193223 (PMC13664985; doi:10.3390/healthcare14193223)
Supplement: Supplementary file 1 [file healthcare-14-03223-s001.zip › Supplementary File S3 English translation.pdf]

Pregnancy Medication Adherence Model (PMAM)
English Questionnaire

English translation of the Romanian study questionnaire for supplementary material

This questionnaire assesses perceptions, concerns, informational and social influences, the relationship with the doctor, and behaviours related to medicine use during pregnancy. Responses are confidential and are used for research purposes.

Participation in this study is voluntary. By completing this questionnaire, you confirm that you have been informed about the purpose of the study and agree to participate.

Section A. Sociodemographic and obstetric information

Age: ________ years

Area of residence: ☐ Urban ☐ Rural

Educational attainment: ☐ Lower secondary ☐ Upper secondary ☐ Post-secondary ☐ University ☐ Postgraduate

Pregnancy trimester: ☐ I ☐ II ☐ III

Section B. Perceptions and factors associated with medicine use during pregnancy – Q5–Q24

Response scale: 1 = Strongly disagree … 5 = Strongly agree

Item	1	2	3	4	5
Q5. I understand why the treatment was prescribed.	☐	☐	☐	☐	☐
Q6. I understand the benefits of the treatment.	☐	☐	☐	☐	☐
Q7. Patient information leaflets make me think that the treatment is dangerous.	☐	☐	☐	☐	☐
Q8. I find it difficult to distinguish which medicines are safe.	☐	☐	☐	☐	☐
Q9. I understand the risks of not taking the treatment.	☐	☐	☐	☐	☐
Q10. I am afraid that the treatment may harm my baby.	☐	☐	☐	☐	☐
Q11. I feel anxious when taking medicines.	☐	☐	☐	☐	☐
Q12. I prefer to avoid treatment.	☐	☐	☐	☐	☐
Q13. My fear is stronger than my trust.	☐	☐	☐	☐	☐
Q14. I feel more reassured after my doctor’s explanations.	☐	☐	☐	☐	☐
Q15. I look for information about medicines on the internet.	☐	☐	☐	☐	☐
Q16. Social media influences my decisions.	☐	☐	☐	☐	☐
Q17. My family influences my decisions.	☐	☐	☐	☐	☐
Q18. The experiences of other pregnant women matter to me.	☐	☐	☐	☐	☐
Q19. My family’s support helps me follow the treatment.	☐	☐	☐	☐	☐

Item	1	2	3	4	5
Q20. I trust my doctor.	☐	☐	☐	☐	☐
Q21. My doctor explained the treatment clearly.	☐	☐	☐	☐	☐
Q22. I felt listened to.	☐	☐	☐	☐	☐
Q23. My relationship with my doctor helps me follow the treatment.	☐	☐	☐	☐	☐
Q24. Clearer explanations would help me follow the treatment.	☐	☐	☐	☐	☐

Section C. Adherence and non-adherence behaviours – Q25–Q28

Response scale: 1 = Never … 5 = Always

Reference period for Q25–Q28: consider any medicine prescribed during the current pregnancy up to the time you complete this questionnaire.

Item	1	2	3	4	5
Q25. I followed the treatment as instructed.	☐	☐	☐	☐	☐
Q26. I forgot to take my medicines.	☐	☐	☐	☐	☐
Q27. I stopped the treatment without medical approval.	☐	☐	☐	☐	☐
Q28. I avoided starting the treatment because of fear.	☐	☐	☐	☐	☐

Section D. Open-ended question

Q29. What is your main fear regarding the use of medicines during pregnancy?

____

____

____

____

____

Methodological note

Note: Q5–Q24 assess perceptions and potential factors associated with adherence. Q25 is the primary self-reported adherence item, whereas Q26–Q28 assess non-adherence-related behaviours. Q29 is an open-ended question. Negatively worded items are reverse-coded only when composite scores are calculated; raw responses remain unchanged in the questionnaire. This document reproduces the questionnaire items used in the study; its inclusion as supplementary material does not itself establish validation of a clinical instrument.

This document is an English translation of the Romanian study questionnaire. It has not undergone separate linguistic or cross-cultural validation and should not be interpreted as a validated English-language version.
